# Enhanced astigmatism correction accuracy with image-guided toric ICL implantation: a comparative clinical analysis with manual axis marking

**DOI:** 10.3389/fmed.2026.1672270

**Published:** 2026-02-27

**Authors:** Ruixia Li, Zheng Fu, Yang Liu, Caiyuan Liu, Jianzhang Hu

**Affiliations:** 1 The Graduate School of Fujian Medical University, Fuzhou, China; 2 School of Medicine, Xiamen University Affiliated Xiamen Eye Center, Eye Institute of Xiamen University, Xiamen University, Xiamen, Fujian, China; 3 Department of Ophthalmology, Fujian Medical University Union Hospital, Fuzhou, China

**Keywords:** Alpins vector analysis, astigmatism, image-guided, manual marking, TICL

## Abstract

**Purpose:**

This study compared clinical outcomes of toric intraocular collamer lens (TICL) implantation for myopia and astigmatism correction using image-guided navigation versus manual astigmatic marker alignment, evaluating postoperative astigmatism correction accuracy.

**Methods:**

A retrospective analysis included 250 patients (467 eyes) undergoing TICL implantation between January and June 2023. Patients were divided into manual (slit-lamp axis marking) and image-guided (navigation-adjusted lens positioning) groups. Preoperative parameters were comparable. Postoperative visual acuity, refraction, and astigmatism vector analysis (TIA, SIA, DV, CI, AE) were assessed at 3 months.

**Results:**

No significant differences were observed in preoperative parameters, postoperative spherical equivalent (SE), or corrected distance visual acuity (CDVA) (*P* > 0.05). However, sphere, cylinder, and uncorrected distance visual acuity (UDVA) differed significantly (*P* < 0.05). Vector analysis revealed no intergroup difference in target-induced astigmatism (TIA) (*P* > 0.05), but surgically induced astigmatism (SIA), absolute difference vector (DV), correction index (CI), and nd absolute Angle of Error (AE) showed significant improvements in the image-guided group (*P* < 0.05). Arithmetic AE did not differ between groups (*P* > 0.05). No intraoperative/postoperative complications (e.g., corneal edema, iris prolapse) occurred.

**Conclusion:**

Image-guided TICL implantation yielded superior visual outcomes and greater astigmatism correction accuracy compared to manual marking, with no complications reported. This highlights the clinical advantage of navigation systems in optimizing refractive precision.

## 1 Introduction

Currently, artificial lens implantation surgery for crystalline lens eyes has been widely used in refractive surgery, and many studies have shown its safety and stability. The effectiveness of TICL implantation in correcting astigmatism in patients has also been confirmed (1–3).

For patients with astigmatism, the accuracy of the implanted lens position directly affects the postoperative visual quality and even affects the postoperative visual acuity. Currently, most of the global preoperative marking of the lens relies on manual marking (4). If the patient’s cooperation during manual marking is poor or the doctor’s operation is not proficient, there may be large errors, affecting the accuracy and predictability of astigmatism correction. Literatures found that the Callisto Eye image-guided system has been recognized by experts and scholars for its accuracy in astigmatic lens implantation in cataract patients (5–8), but there are few reports on whether the Callisto Eye navigation system is more accurate in TICL implantation surgery at home and abroad.

This retrospective study reviewed the use of image-guided system for positioning in TICL implantation surgery, to obtain more accurate and effective astigmatism correction than manual marking, which will guide us to use image-guided system more in clinical practice.

## 2 Materials and methods

### 2.1 Study design and patient selection

A retrospective study encompassed 250 patients (467 eyes) who underwent TICL lens implantation at our institution from January 2023 to June 2023.

Patients aged ≥ 18 years who underwent TICL implantation were eligible for inclusion if they had stable refraction, myopia ≤ −18.0 diopters, regular corneal astigmatism between 0.50 and 6.0 diopters, with an anterior chamber depth ≥ 2.8 mm and endothelial cell density ≥ 2,000 cells/mm^2^, with a clear crystalline lens. Patients were excluded if they had a history of prior intraocular or corneal refractive surgery, glaucoma or ocular hypertension, corneal pathology including keratoconus or corneal ectasia, visually significant cataract, retinal pathology affecting visual outcomes, or other ocular conditions that could influence postoperative refractive results.

Among them, there were 68 male patients (27.2%) and 182 female patients (72.8%), with ages ranging from 18 to 42 years. The patients were divided into manual and image-guided groups based on the preoperative marking method for the TICL lens astigmatism axis. The preoperative choice of manual or navigation was random. This study was conducted following informed consent from all patients and approval from the ethics committee.

### 2.2 Preoperative examination

Preoperative assessments included unaided visual acuity, best-corrected visual acuity, manifest refraction, corneal topography, corneal endothelium, Optos fundus photography, anterior and posterior segment OCT, UBM (Ultrasoundbiomicroscopy), CASIA2 (TOMEY) and IOLMaster700 (Carl Zeiss Meditec, AG). In the image-guided group, the IOLMaster700 examination results were transferred to the Callisto Eye navigation system in the operating room.

### 2.3 Surgical technique

**FIGURE 1:**
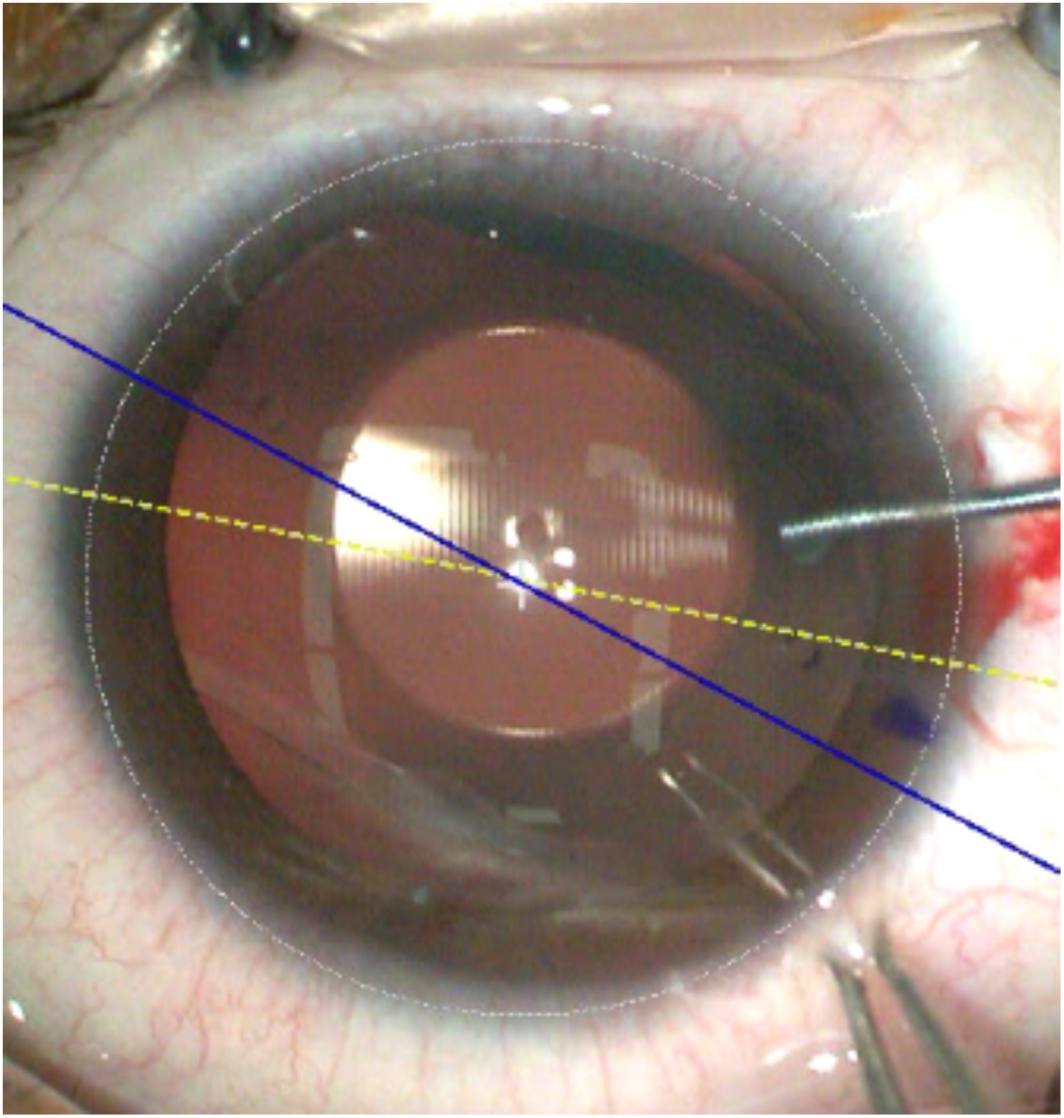
There is a discrepancy between the manual marking point and the blue line indicated by the image-guided system.

All surgeries were performed by the same experienced surgeon. Three days before surgery, the patients were instructed to apply Levofloxacin eye drops (0.5%, Santen Pharmaceutical, Japan) four times a day to the operated eye to prevent infection. The conjunctival sac was rinsed preoperatively, and under topical anesthesia, the lens rotation axis was manually marked under a slit lamp microscope as follows: The slit lamp’s light band was adjusted to the rotation axis, and a line was drawn along the corneal edge using a 1 ML syringe needle. Subsequently, the area was colored with a marking pen. Mydriasis was induced using compound tropicamide eye drops (Mydrin-P, Santen Pharmaceutical) to a size of 7–8 mm. Standard draping and disinfection procedures were followed, and the TICL was inserted into the anterior chamber, and preloaded into the artificial lens chamber. A 2.8 mm blade was used to create a three-step incision on the temporal corneal edge, progressing along the corneal meridian toward the anterior chamber, ensuring a slow insertion and natural unfolding of the TICL. Care was taken to adjust the angle during insertion to prevent contact with the corneal endothelium and crystalline lens. Viscoelastic was injected anterior to the artificial lens to maintain appropriate anterior chamber depth. In the manual group, the TICL’s four footplates were positioned within the ciliary sulcus using a positioning hook, aligning the axis rotation with the preoperative mark. In the image-guided group, alignment was achieved by matching the real-time image with the blue line indicating the rotation axis on the image-guided system, allowing for observation of any discrepancies between the preoperative manual marks and the navigation’s blue line indication (Figure 1). After positioning, the viscoelastic in the anterior and posterior chambers was cleared, ensuring gentle movements to avoid significant intraocular pressure fluctuations, iris prolapse, or lens displacement. Intraocular pressure and lens positioning were then observed, with the manual group corresponding to the preoperative mark and the navigation group aligning with the real-time image’s rotation axis. Levofloxacin eye drops were instilled in the conjunctival sac, and a transparent eye shield was placed over the operated eye. Routine intraocular pressure checks and anterior chamber assessments were conducted 3–5 h postoperatively, with standard antibiotic eye drops administered. All surgeries in this study were successfully completed.

### 2.4 Postoperative follow-up

Outpatient follow-up visits were scheduled on postoperative day 1, day 7, 1 and 3 months. Spherical error, cylindrical error, spherical equivalent (SE), uncorrected distance visual acuity (UDVA), corrected distance visual acuity (CDVA), expressed in logarithm of the minimum angle of resolution (logMAR), and intraocular pressure were monitored.

### 2.5 Vector analysis

Astigmatism involves axis orientation, therefore the Alpins vector analysis method was used to analyze astigmatism changes and evaluate postoperative outcomes (9–12).

Target induced astigmatism (TIA) represents the astigmatism vector magnitude required for surgery, indicating the vector difference between preoperative astigmatism and expected remaining astigmatism. SIA (surgically induced astigmatism) denotes the actual astigmatism vector introduced during surgery. DV (difference vector) represents the vector difference between the surgically induced astigmatism (SIA) and the target induced astigmatism (TIA), reflecting the magnitude of resultant astigmatism. CI (correction index) is defined as the ratio of SIA to TIA, with a value of 1 indicating ideal correction, values greater than 1 indicating overcorrection, and values less than 1 indicating undercorrection. Angle of Error was calculated using the Alpins method as the angular difference between the axes of surgically induced astigmatism and target-induced astigmatism. Arithmetic Angle of Error retained the direction of deviation, whereas absolute Angle of Error was defined as the absolute value of this difference, folded into the 0–90° range. When AE > 0, it indicates that the surgical correction of astigmatism is counterclockwise to the expected correction. When AE < 0, it indicates that the surgical correction of astigmatism is clockwise to the expected correction.

### 2.6 Statistical analysis

Statistical processing and analysis were carried out using SPSS 25.0 software. Continuous refractive and visual acuity variables are presented as mean ± standard deviation when normally distributed and as median (interquartile range) when non-normally distributed. Between-group comparisons were performed using independent-samples *t*-tests. Generalized estimating equations with an exchangeable correlation structure were applied to compare postoperative refractive and astigmatism vector outcomes between groups, accounting for clustering by patient. *P* < 0.05 was considered statistically significant.

## 3 Results

A total of 250 patients (467 eyes) were included, comprising 175 patients in the manual marking group and 75 patients in the image-guided group. Baseline demographic and preoperative characteristics are summarized in Table 1. Of these, 217 patients contributed both eyes and 33 contributed one eye. In the manual marking group, 46 patients were male and 129 were female, while the image-guided group included 22 male and 53 female patients. The median age was 26 years (interquartile range, 22–29 years) in the manual marking group and 26 years (interquartile range, 22–31 years) in the image-guided group.

**TABLE 1:** Comparison of the visual and refractive parameters for manual groups versus the image-guided groups in 467 eyes.

Parameter	Manual groups	Image-guided groups	*P*-value
Preoperative spherical error (D), mean ± SD	−7.68 ± 2.24	−7.54 ± 2.42	0.561
Preoperative cylindrical error (D), median (IQR)	−2.00 (−2.50 −1.25)	−1.50 (−2.25 −1.50)	0.368
Preoperative spherical equivalent (D), mean ± SD	−8.65 ± 2.26	−8.48 ± 2.48	0.476
Postoperative spherical error (D), mean ± SD	0.27 ± 0.51	0.12 ± 0.47	0.020
Postoperative cylindrical error (D), median (IQR)	−0.50 (−0.75 to 0.50)	−0.25 (−0.50 to 0.00)	<0.001
Postoperative spherical equivalent (D), mean ± SD	−0.11 ± 0.39	−0.00 ± 0.49	0.018
Preoperative uncorrected distance visual acuity (UDVA) (logMAR), mean ± SD	1.24 ± 0.37	1.27 ± 0.36	0.623
Postoperative uncorrected distance visual acuity (UDVA) (logMAR), mean ± SD	−0.02 ± 0.08	−0.06 ± 0.06	<0.001
Preoperative corrected distance visual acuity (CDVA) (logMAR), mean ± SD	0.03 ± 0.08	0.02 ± 0.08	0.313
Postoperative corrected distance visual acuity (CDVA) (logMAR), mean ± SD	−0.05 ± 0.07	−0.049 ± 0.06	0.897

Postoperatively, the image-guided group demonstrated significantly lower residual spherical error (*P* = 0.020), cylindrical error (*P* < 0.001), and spherical equivalent (*P* = 0.018) compared with the manual marking group. Postoperative uncorrected distance visual acuity was also significantly better in the image-guided group (*P* < 0.001). In contrast, postoperative corrected distance visual acuity did not differ significantly between groups (*P* = 0.897).

### 3.1 Astigmatism vector analysis

**FIGURE 2:**
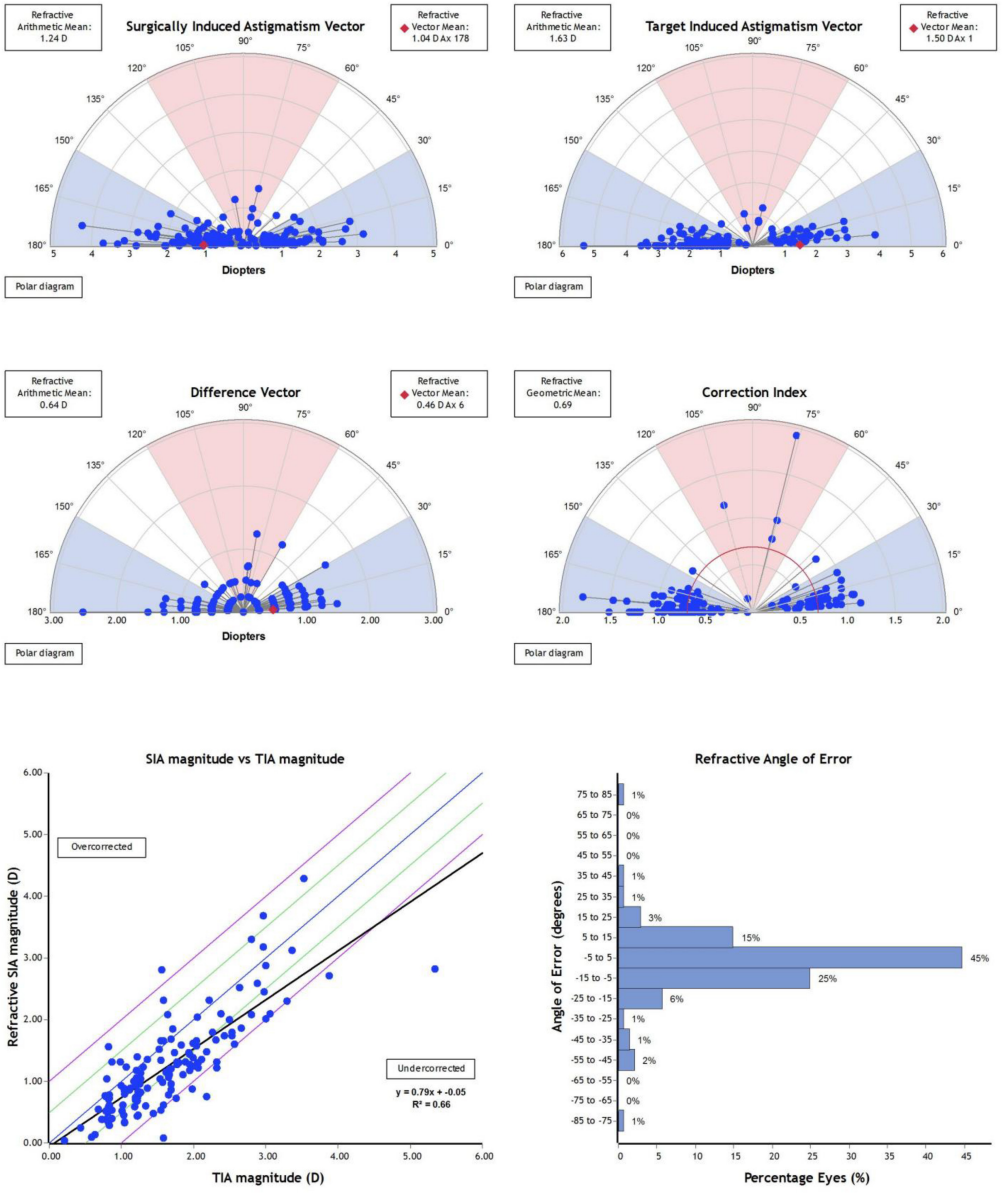
The distribution of vector analysis parameters in the manual group.

**FIGURE 3:**
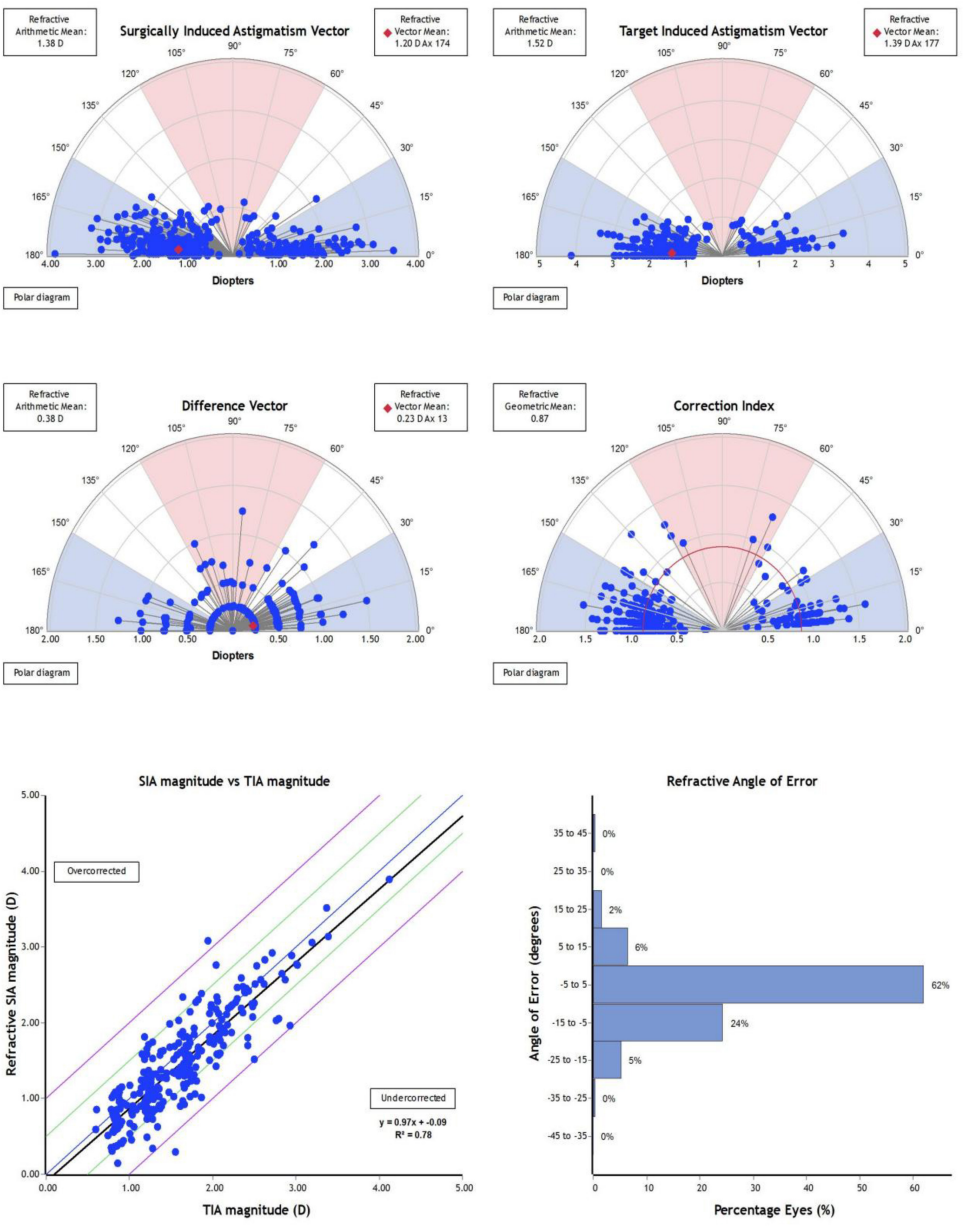
The distribution of vector analysis parameters in the navigation group.

The distribution of vector analysis parameters between the manual and image-guided groups can be seen in Figures 2, 3. Astigmatism vector outcomes were compared between groups using generalized estimating equations with clustering by patient (Table 2). Target-induced astigmatism did not differ between the manual marking and image-guided groups (*P* = 0.146). The image-guided group showed greater accuracy of astigmatic correction, with a smaller difference vector (*P* < 0.001) and a correction index closer to unity (*P* < 0.001). There was no between-group difference in arithmetic angle of error (*P* = 0.95); however, absolute angle of error was lower in the image-guided group (*P* < 0.001), indicating reduced rotational misalignment. Using predefined refractive criteria, resultant astigmatism consistent with rotational misalignment was identified based on postoperative refractive cylinder magnitude and deviation between the postoperative refractive axis and the intended treatment axis. Eyes with a postoperative refractive cylinder of at least 0.75 D and an absolute axis deviation of at least 5° met these criteria. Accordingly, 23 of 141 eyes (16.3%) in the manual group and 32 of 326 eyes (9.8%) in the image-guided group demonstrated resultant astigmatism consistent with axis-related error.

**TABLE 2:** Alpins vector analysis outcomes after toric implantable collamer lenses (ICL) implantation in 467 eyes.

Outcome	Manual [median (IQR)]	Image-guided [median (IQR)]	Estimated mean difference (95% CI)	*P*-value
Target-induced astigmatism (D)	1.54 (1.04–2.04)	1.36 (1.18–1.82)	−0.11 (−0.25, 0.04)	0.146
Surgically induced astigmatism (D)	1.11 (0.73–1.59)	1.26 (0.91–1.73)	0.14 (−0.00, 0.28)	0.056
Difference vector (D)	0.51 (0.48–0.76)	0.25 (0.25–0.50)	−0.26 (−0.33, −0.19)	<0.001
Correction index	0.72 (0.60–0.87)	0.89 (0.79–1.00)	0.15 (0.10, 0.20)	<0.001
Arithmetic angle of error (°)	−0.55 (−8.31 to 3.46)	−2.03 (−5.81 to 0.00)	0.09 (−2.64, 2.81)	0.950
Absolute angle of error (°)	5.77 (2.47–11.04)	3.46 (1.30–7.55)	−4.26 (−6.47, −2.05)	<0.001

## 4 Discussion

**FIGURE 4:**
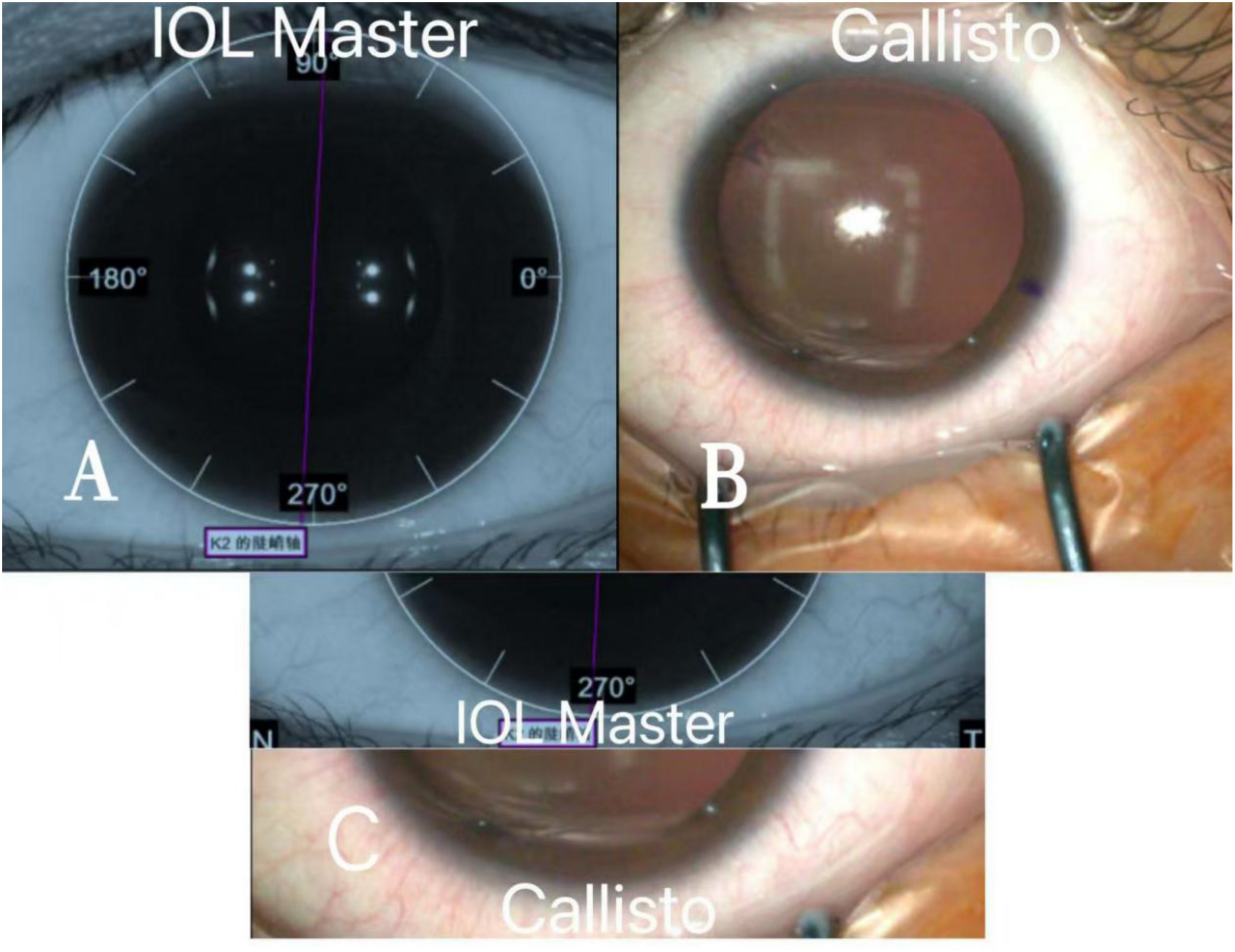
Illustration of the image-guided system matching preoperative conjunctival vascular images of patients with intraoperative microscopic conjunctival vascular images. **(A)** Shows the ocular image captured during the preoperative seated IOL master examination of the patient. **(B)** Shows a real-time snapshot of the patient’s ocular image under the microscope during surgery. **(C)** Demonstrates the navigation system aligning and positioning the location of ocular conjunctival vessels by comparing the preoperative ocular images with real-time intraoperative images.

In the selection of surgical methods for lens extraction, the implantation of artificial lenses in individuals with crystalline eyes has traditionally served as a supplementary procedure for corneal refractive surgery. The primary candidates for this surgery are patients with thin corneal or high degrees of refractive unsuitable for corneal laser surgery. However, in recent years, the safety and efficacy of ICL implantation in low to moderate degrees of refractive error have gradually gained recognition (13–16). The widespread application of ICL lens implantation has followed suit. The main focus after TICL surgery is the rotation of the intraocular lens and the resultant diopter (4). Precise positioning of astigmatic lenses is a critical step prone to errors. The main sources of error in manual positioning under a slit lamp include: ➀ Patient sensitivity and involuntary movement, leading to inaccurate positioning due to the inability to keep the eye open and focused for a certain period. ➁ Difficulty in marking the upper position point for vertically placed lenses due to the upper eyelid’s coverage, necessitating secondary marking during surgery, thereby increasing errors. ➂ The change in position from sitting to lying during can affect the accuracy of lens placement. ➃ Poor surgical proficiency or the influence of handedness. ➄ The thickness and clarity of the marking pen can affect visibility, and the rinsing of the conjunctival sac during tear secretion or disinfection can cause the color of the mark to fade or disappear, making it difficult to accurately identify the marked points. The image-guided system, utilizing images of the conjunctival vessels captured by the IOL Master 700 while the patient is seated, imported into the system, and matched with the vessels during surgery while the patient is lying down, eliminates the influence of manual errors, reduces dependence on the physician, and offers higher accuracy (Figure 4).

In this study, image guided toric ICL implantation was not associated with an increased incidence of surgical complications when compared with manual axis marking, indicating comparable procedural safety. Moreover, the navigation group achieved superior postoperative uncorrected visual acuity results, and sphere, cylinder and UDVA demonstrated statistical significance, indicating that the astigmatism correction in the navigation group is more meaningful for improving postoperative uncorrected visual acuity in patients with refractive errors. In the results, there was no difference in TIA between the two groups. However, SIA, DV, and CI showed statistical significance between the two groups (*P* < 0.05), suggesting that the navigation group had a better astigmatism correction effect with better precision. This is consistent with previous reports indicating higher accuracy in astigmatism correction with navigation in cataract astigmatism lens implantation (17–19). Notably, arithmetic AE did not differ between groups, indicating the absence of a systematic directional bias in axis placement, whereas absolute Angle of Error was significantly smaller in the image-guided group, reflecting reduced rotational deviation. Angle of Error, therefore, remains the primary parameter for quantifying rotational error. Difference vector, in contrast, represents the resultant vector discrepancy between the intended and achieved astigmatic correction and serves as a summary scorecard of precision rather than a direct measure of surgical error. Accordingly, the smaller difference vector observed in the image-guided group indicates improved overall precision of astigmatic correction. Correction index, which reflects the accuracy of magnitude correction alone and is independent of axis alignment, was closer to unity in the image-guided group, indicating more accurate correction magnitude despite a tendency toward undercorrection in both groups. Remaining astigmatism at 3 months postoperatively, measured as postoperative refractive cylinder, was present in both groups. This finding likely reflects the combined effects of resultant vector precision and other factors not captured by vector analysis alone.

This study represents a preliminary evaluation of short-term astigmatic outcomes following toric implantable collamer lens implantation using image-guided navigation, with a primary focus on early refractive precision. Detailed postoperative safety parameters such as intraocular pressure, vault, and endothelial cell density were not uniformly available and could not be analyzed. The follow-up period of 3 months is relatively short and therefore precludes conclusions regarding long-term rotational stability. Previous longitudinal studies have reported that most postoperative astigmatic change and lens rotation occur during the early postoperative period, with no statistically significant differences observed between short- and longer-term follow-up intervals, such as between 3 and 6 months or between 2 months and 2 years after surgery (20, 21). Long-term studies with extended follow-up are warranted to further assess the stability of astigmatic correction. In addition, the retrospective design and the imbalance in sample size between groups represent limitations that may have influenced the results. Although postoperative refractive outcomes were used to estimate the proportion of eyes with resultant astigmatism consistent with axis-related error, postoperative toric ICL axis orientation was not directly measured, and these estimates should therefore be interpreted as indirect rather than definitive evidence of correctable lens rotation.

In conclusion, the navigation system is convenient and accurate. Its application reduces patient fear of needles during manual preoperative marking, saves time by eliminating prolonged preparation due to poor patient cooperation during marking, and most importantly, effectively reduces errors in manual axis marking. TICL lens implantation under navigation guidance is safe and offers more precise astigmatism correction compared to manual marking.

## Data Availability

The original contributions presented in this study are included in this article/supplementary material, further inquiries can be directed to the corresponding author.
